# Effectiveness and Adherence of Dry Eye Patients Who Switched from Short- to Long-Acting Diquafosol Ophthalmic Solution

**DOI:** 10.3390/jcm12134495

**Published:** 2023-07-05

**Authors:** Sho Ishikawa, Takanori Sasaki, Takahumi Maruyama, Koichiro Murayama, Kei Shinoda

**Affiliations:** 1 Department of Ophthalmology, Saitama Medical University, Saitama 350-0495, Japanshinok@saitama-med.ac.jp (K.S.); 2 Tsuruse Murayama Eye Clinic, Saitama 354-0021, Japan

**Keywords:** diquafosol ophthalmic solution, dry eye, long active, polyvinylpyrrolidone, P2Y2 receptor agonist

## Abstract

Long-acting (lasting extend) diquafosol ophthalmic solution 3% (DQSLX) is administered three times daily versus six times daily for the currently approved diquafosol ophthalmic solution (DQS). We investigated the efficacy and adherence of switching from DQS to DQSLX in patients with dry eye disease. We retrospectively enrolled 54 patients (17 men and 37 women) with eye drop prescription changes from DQS to DQSLX between December 2022 and March 2023. The number of eye drops, subjective symptoms, tear breakup time (TBUT), and fluorescein staining scores from baseline to 4 weeks after starting DQSLX were evaluated. Participants then chose between DQSLX and DQS. Patients administered DQSLX three times per day, as listed on the package insert, 88.9% of the time; significantly higher than the 5.6% of patients who used DQS six times per day, as instructed. The DQSLX group showed significant improvements in symptoms and fluorescein staining scores (23.3 ± 20.1 and 0.8 ± 1.7, respectively) compared with the baseline (37.8 ± 24.1 and 1.1 ± 1.5, *p =* 0.01 and <0.001, respectively). The TBUT in the DQSLX group (5.0 ± 2.5 s) did not significantly improve compared to the DQS group (4.5 ± 1.7 s) (*p =* 0.75). Fifty-one (94.4%) patients opted to continue DQSLX because of the pleasant feeling of the eye drops, long-lasting moisture, and less frequent administration. The efficacy and adherence of DQSLX was comparable to DQS.

## 1. Introduction

Dry eye disease is a multifactorial condition of tear film instability that produces a range of discomforting ocular symptoms and/or visual impairment, with potentially damaging effects on the ocular surface [1,2]. Dry eye workshop II (DEWS II) proposes diagnostic tests to examine dry eye disease, including subjective symptoms, tear breakup time (TBUT), tear osmolarity, and ocular surface staining. It also evaluates abnormal lipids, meibomian gland dysfunction (MGD), and tear volume as subtype classification tests [1]. Dry eyes are classified into the tear volume deficiency and evaporative types. Therapeutic options for treating tear deficiency-type dry eye disease include artificial tears, anti-inflammatory therapy, secretagogues, and tear retention treatment [3]. The Asia Dry Eye Society stresses the instability of the tear film and the importance of visual impairment, highlighting an essential role for TBUT assessment [2]. Tear film instability is caused by damage to one or more of the three layers of the tear film, which are the mucin, aqueous, and/or lipid layers. The Asia Dry Eye Society advocates the treatment of dry eye with Tear Film Oriented Therapy-TFOT, which targets the three layers of the tear film (mucin, aqueous, and lipid) [2].

Diquafosol ophthalmic solution (DQS) is a purinergic P2Y2 receptor agonist approved for treating DED in Japan. Diquafosol is a 3% ophthalmic solution that stimulates tear fluid and mucin secretion [4,5]. It is reported that DQS may increase the function of the tear film lipid layer by promoting spreading of the lipid layer through lipid and tear fluid secretion [2]. In randomized, double-blind, multicentre trials in patients with dry eye, significantly greater improvements in fluorescein and rose bengal staining scores were seen with DQS than with placebo, and DQS was noninferior to sodium hyaluronate ophthalmic solution 0.1%, in terms of the improvement in the fluorescein staining score, and more effective than sodium hyaluronate ophthalmic solution 0.1%, in terms of the improvement in the rose bengal staining score [6,7,8,9]. The efficacy of DQS in the treatment of dry eye was maintained in the longer term, with improvements also seen in subjective dry eye symptoms, and was also shown in a real-world setting [4]. Diquafosol ophthalmic solution 3% also demonstrated efficacy in various specific dry eye disorders, including aqueous-deficient dry eye, short tear film break-up time dry eye, and obstructive meibomian gland dysfunction [4,5,6,7,8,9].

Despite the efficacy of DQS, poor compliance with the number of eye drops administered is problematic. Although the package insert states that DQS should be administered six times a day, Uchino et al. reported that only 8.3% of patients administered DQS six times daily [10]. It has been reported that those who used a fixed number of eye drops had improved subjective symptom scores compared to those who used drops on an as-needed basis [10,11]. Patients using DQS may not experience the full benefits due to insufficient frequency.

In November 2022, a long-acting (lasting) 3% diquafosol ophthalmic solution, (DQSLX), was approved in Japan. DQSLX successfully extended the effect time using polyvinylpyrrolidone (PVP) as an additive. PVP has been reported to exhibit electrostatic interactions with secretory mucins, membrane mucins, and water [12,13]. DQSLX is thought to work concurrently with PVP for prolonged effect duration: DQS increases mucin secretion, and PVP–mucin binding promotes retention of tear fluid on the ocular surface, which prolongs their duration of action. DQSLX requires administration three times a day—fewer than the six times daily required for DQS. It has been reported to improve corneal and conjunctival staining scores compared to a placebo [14]. However, there are no reports comparing DQSLX and DQS.

In the present study, we retrospectively compared the changes in eye drop use, TBUT, and fluorescein staining scores in patients who switched from DQS to DQSLX.

## 2. Materials and Methods

### 2.1. Participants

Institutional Review Board/Ethics Committee approval was obtained from the Ethics Committee of Saitama Medical University Hospital (2023-020). This study adhered to the tenets of the Declaration of Helsinki. The need for written informed consent was waived by the ethics committee of Saitama Medical University due to the retrospective design of the study.

We retrospectively enrolled 54 patients (17 men, 37 women) whose eye drop prescription was changed from DQS to DQSLX at Saitama Medical University Hospital, Hokumou Hospital, or Tsuruse Murayama Eye Clinic between December 2022 and March 2023. Patients with any of the following criteria were excluded: use of alternative dry eye drops, use of DQS drops for shorter than 3 months, use of over-the-counter dry eye medications, use of lubricating drops, use of oral medications to increase tear fluid volume (e.g., pilocarpine), comorbidities including diabetes or ocular infections, those who had glaucoma or allergic conjunctivitis treated by eye drop instillation, those who had undergone ophthalmic surgery within 1 month, those who had a history of punctual plug or surgical procedure for dry eye, and lack of informed consent.

In all subjects, we first assessed the subjective symptoms of dry eye from the medical records. All clinics and hospitals used symptom assessment in dry eye (SANDE) routinely for the assessment of subjective symptoms of dry eye [15]. The SANDE is a short and intuitive questionnaire based on a visual analog scale that quantifies the severity and frequency of dry eye symptoms, including dryness and irritation. It comprises two questions, and each employs a 100 mm horizontal linear visual analog scale. The SANDE scores ranged from 0 to 100. The average value of dryness and irritation was used for statistical analysis. We also collected data on the frequency of administration. Then, we asked questions about the frequency of instillation per day for the treatment of DQS in the last 1 month. The number of times of eye drops was selected from the following options as the most applicable: 0, 1, 2, 3, 4, 5, 6, 7, and 8 times or more.

Thereafter, we assessed the dry eye parameters, fluorescein staining scores, and TBUT. Vital staining was performed as follows: A preservative-free solution (2 µL) containing 1% fluorescein dye was instilled into the conjunctival sac using a micropipette. Fluorescein staining scores were assigned on a scale of 0–9, [16,17]. Fluorescein TBUT was measured using a fluorescein solution without anesthesia. The participants were instructed to blink several times to ensure adequate mixing of the fluorescein dye into the tear film. The interval between the last complete blink and the appearance of the first corneal black spot in the stained tear film was measured three times, and the average value was included for statistical analysis. Then, we classified into sub types of dry eye with tear-film-oriented diagnosis (TFOD) [18]. TFOD is a diagnostic method for dry eye based on the tear film dynamics and breakup patterns, through which dry eye subtypes, including aqueous-deficient dry eye, decreased-wettability dry eye, and increased-evaporation dry eye, are diagnosed. We classified by categorizing the fluorescein breakup patterns into five types in this study. Area break (AB) occurs when the aqueous tear volume is extremely diminished. Line break (LB) results from the simultaneous action between the drag of the aqueous tear by the spreading tear lipid layer and suction effects on the aqueous tear from the lower tear meniscus. Spot break (SB) and dimple break (DB) are considered to result from the impaired wettability of the corneal surface. Random break (RB) is considered to be related to increased evaporation.

We compared subjective symptoms and objective findings before and four weeks after using the DQSLX. Four weeks after using DQSLX, patients were asked to choose whether they would continue to use DQSLX or DQS eye drops, and the reasons for this were extracted from their medical records.

### 2.2. Statistical Analyses

All statistical analyses were performed using JMP version 17^®^ software (SAS Institute, Tokyo, Japan). All data are expressed as means ± standard deviation. The Wilcoxon signed-rank test was used to compare the SANDE score, fluorescein staining score, and TBUT before versus after 4-week DQSLX. Superiority of DQS or DQSLX was determined using a Fisher’s exact test. All analyses of the objective findings used values from the right eye. Statistical significance was set at *p* < 0.05.

## 3. Results

**Figure 1:**
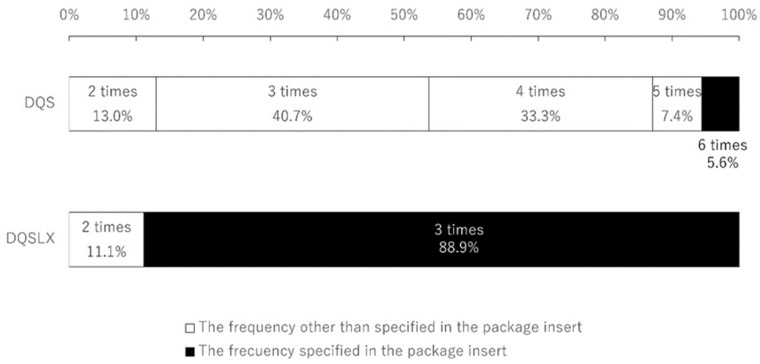
Distribution of actual eye drop frequency.

A total of 54 patients (54 eyes, 17 men and 37 women) were enrolled in this study, and the average patient age was 59.4 ± 16.0. There was no aqueous-deficient dry eye patient with Sjögren syndrome. Only 3 patients (5.6%) were using DQS six times per day as instructed; the average frequency of DQS administration was 3.5 ± 1.0. In contrast, 48 patients (88.9%) were using DQSLX three times per day as instructed; the average frequency of DQSLX administration was 2.9 ± 0.3 (Figure 1). Significantly more patients used DQSLX at the frequency instructed on the package insert than used DQS as instructed (*p* < 0.001). No patient answered to more than seven times per day of eye drops or zero times per day of eye drops. We asked patients how they ensured the number of times they instilled DQS was six times a day, and whether they set an alarm on their digital devices (1 patient used a computer and 2 patients used an application of a smartphone) to keep track of the number of times they used the drops.

DQS administration is listed on the package insert as six times a day, but only 5.6% of patients used it six times a day. DQSLX use was listed on the package insert as three times a day, and 88.9% of the patients used DQSLX three times a day.

Classifications of TBUT patterns were made in all patients. In total, 30 eyes (55.6%) were classified into RB, followed by 14 eyes (25.9%) into DB, and 10 eyes (18.5%) into LB. There was no patient classified into AB or SB.

At week 4 after commencement, the DQSLX group showed significant improvements in symptoms (SANDE score) and fluorescein staining scores (23.3 ± 20.1 and 0.8 ± 1.7, respectively) compared to baseline (37.8 ± 24.1 and 1.1 ± 1.5, *p =* 0.01 and <0.001, respectively). The TBUT at week 4 in the DQSLX group (5.0 ± 2.5 s) did not significantly improve compared to baseline (4.5 ± 1.7 s) (*p =* 0.75) (Table 1). In the subtype of dry eye, the DQSLX group showed significant improvements in symptom score in RB and in DB (*p =* 0.01 and 0.04, respectively), but did not significantly improve in LB. The DQSLX group did not significantly improve TBUT in RB, DB or LB (*p =* 0.32, 0.67 and 0.60, respectively). The DQSLX group showed significant improvements in fluorescein staining scores in DB and LB (*p* < 0.001 and <0.001, respectively), but not significantly in RB (*p =* 0.06) (Table 2). At week 4 after commencement, 49 eyes (90.7%) did not change TBUT patterns, 3 eyes (5.6%) changed from DB to RB, 1 eye (1.9%) changed from LB to RB, and 1 eye (1.9%) changed from DB to LB.

At week 4, 94.4% of the DQSLX patients chose DQSLX. DQSLX was chosen due to the pleasant feeling of the eye drops and long-lasting moisture. Conversely, side effects were the main reason for not choosing DQSLX, including the appearance of discharge (n = 1), exacerbation of discharge (n = 1), and strong sticky feeling with irritation (n = 1). Two patients discontinued the use of DQS because of increased discharge.

## 4. Discussion

In our study, we demonstrated that patients using DQS for >3 months had lower eye drop adherence, which was significantly improved by switching to DQSLX. At week 4, the DQSLX group showed significant improvements in subjective symptoms (SANDE score) and fluorescein staining scores, compared to baseline. Fifty-one (94.4%) patients opted to continue DQSLX because of the pleasant feeling of the eye drops, long-lasting moisture, and less frequent administration. Adverse drug reactions included eye discharge (3.6%) and eye irritation (1.8%) at week 4 in the DQSLX group.

The addition of PVP differentiates DQSLX and DQS. PVP was first used from the 1950s as a blood plasma expander for trauma victims and is now used in many pharmaceutical tablets and liquid formulations [19]. PVP has been reported to exhibit electrostatic interactions with secretory mucins, membrane mucins, and water [12,13]. DQS has been shown to stimulate water secretion from conjunctival epithelial cells and mucin secretion from conjunctival goblet cells via P2Y2 receptors [20,21]. DQSLX is thought to work concurrently with PVP for prolonged effect duration: DQS increases mucin secretion, and PVP–mucin binding promotes retention of tear fluid on the ocular surface, which prolongs their duration of action.

Poor adherence to medical treatment is a common issue in medicine, especially in chronic diseases such as arterial hypertension, diabetes, and glaucoma, in which omission of medication can result in clinically significant symptoms [22]. Adherence to glaucoma medications is a fundamental problem, as 24–59% of patients fail to receive the intended or full effect of treatment [23]. In glaucoma patients, the lowest number of medications instilled with the least frequency enhances patient satisfaction, dosing convenience, and adherence [24,25]. Therefore, DQSLX administered three times daily is expected to result in better adherence than DQS administered six times daily. In this study, adherence to DQSLX (88.9%) was better than that to DQS (5.6%). However, there may be a bias as adherence was only monitored for 4 weeks. Uchino reported that the proportion of participants who actually instilled the eye drops for dry eye disease at the specified frequency 1–3 months after starting treatment was low (DQS 5%, Sodium hyaluronate ophthalmic solution 3%, and Rebamipide ophthalmic suspension 14%) [10]. The frequency of Rebamipide ophthalmic suspension eye drops was 4 times per day, as stated in the package insert. The higher adherence of Rebamipide ophthalmic suspension compared to DQS and sodium hyaluronate ophthalmic solution may be due to the lower frequency of eye drops. As adherence worsens over 1 month even with 4 times daily drops, it is possible that adherence to DQSLX may also worsen. Long-term studies are needed to assess adherence to DQSLX.

A phase 3 study on DQSLX reported similar efficacy and safety in patients with dry eyes compared to a placebo [14]. Fluorescein corneal staining scores and lissamine green conjunctival staining scores significantly improved with DQSLX compared to placebo after 2 and 4 weeks. In contrast, the dryness symptom score, TBUT, and Shirmer’s test 1 did not significantly improve with DQSLX compared to the placebo. In this study, the symptom and fluorescein staining scores significantly improved in the DQSLX group compared to the DQS group at week 4. The same results were obtained despite different controls in the placebo and DQS groups [14].

There are two possible reasons for this. First, DQS may not have been as effective because it was not administered as often as specified in the package insert. The average DQS eye drop administration frequency was 3.5 times; approximately half of that specified in the package insert. However, 41 (75.9%) patients who received DQS still with a TBUT < 5 s met the diagnostic criteria for dry eye [2], despite the use of eye drops. In dry eye patients, subjective dry eye symptoms are significantly improved by fixed eye drop use over as-needed use [10,11]. Therefore, it is possible that the frequency of eye drops was low and the eye drops were not fully effective. Second, DQSLX was more effective than DQS. DQSLX is thought to increase mucin levels through the P2Y2 receptor, and the binding of PVP to this secreted mucin is thought to prolong the duration of action as well as the action of mucin on the ocular surface. However, this study was unable to investigate the superiority of DQSLX because few participants (5.6%) in the study used DQS as specified by the package insert. Further research is required in this area.

In the subtype of dry eye, there was no patient in AB and SB. AB consisted mainly of Sjögren syndrome and non-Sjögren type aqueous deficiency, which accompanies aqueous tear deficiency, and SB consisted mainly of short FBUT dry eye, which was accompanied by a decrease in wettability of the corneal surface, which is supported by membrane-associated mucins, especially the longest one, MUC16 [18,26]. The fluorescein corneal staining score in AB is higher than that of LB, SB, DB, and RB, and the TBUT in SB is shorter than that of LB, DB, and RB [26]. It was reported that AB is the most severe aqueous deficiency type of dry eye and SB is the most severe short FBUT of dry eye [16,26]. In this study, we excluded the patents who made use of alternative dry eye drops, use of over-the-counter dry eye medications, use of oral medications to increase tear fluid volume (e.g., pilocarpine), or who had a history of punctual plug or surgical procedure. Because we excluded patients with severe dry eye, we assumed that there were no AB or SB patients. In this study, the fluorescein staining score in RB did not significantly improve with DQSLX compared to baseline. Random breaks were mainly MGD- and CL-related, which were accompanied by an increase in evaporation. It was reported that the fluorescein corneal staining score in RB is lowest among the subtype of dry eye [26]. We consider that the RB had a lower staining score from the baseline and therefore did not differ significantly from 4 weeks after. In this study, the symptoms score in DB did not significantly improve with DQSLX compared to baseline. It was reported that the symptoms score in DB is lowest among the subtype of dry eye [26]. We consider that the DB had a lower symptoms score from the baseline and therefore did not differ significantly from 4 weeks after.

In this study, TBUT did not significantly improve with DQSLX compared to baseline. In previous reports, there was no difference in TBUT for either DQSLX [14] or DQS [7] after 4 weeks of use. DQS has been reported to improve TBUT after 12 months [27]; therefore, TBUT may also improve with long-term use of DQSLX.

In a phase 3 study, 89% of patients who had previously used DQS indicated that DQSLX was more effective [14]. In this study, 94.4% of patients preferred using DQSLX than DQS. The properties of the solution have been reported to vary depending on the lubricating agents added [28]; therefore, PVP possibly affected the patient experience during DQSLX use. Patients chose DQSLX not only because it requires fewer drops to be administered, but also because of its excellent eye-drop feel.

In a phase 3 study, the adverse drug reactions in the DQSLX group were mild eye irritation (3.6%) and eye discharge (1.8%), with similar incidence to previous clinical studies on DQS [14]. In this study, three cases of adverse drug reactions were observed: eye irritation (1.8%) and eye discharge (3.6%). The results were similar to those of the phase 3 study. We speculate that the side effects did not appear before because of the low frequency of DQS eye drop administration. It is likely that the side effects appeared because of the change to DQSLX and compliance with the frequency of eye drops.

In this study, patients with diabetic complications were excluded, and there were no patients with Sjögren’s syndrome. Both diseases were reported to involve a reduction of the sub-basal corneal nerves and associated corneal perception in the development of dry eye [29,30]. It was reported that DQS improved the tear film dry eye parameters and reduced the symptoms of dry eye in diabetic dry eye patients [31]. In Sjögren’s syndrome, DQS improved the corneal sub-basal nerve fiber density and morphological abnormality of nerve fibers, which was detected with laser-scanning confocal microscopy [30]. DQSLX may also have a more sustained effect on improving corneal perception than DQS, but this could not be explored in this study. Further research is expected.

This study had some limitations. First, the sample size was small, which may have caused bias. In this study, we recruited patients using only DQS and excluded those using multiple eye solutions. Therefore, patients with severe dry eye may have been excluded, which may have affected the adherence results. Data were collected from primary and tertiary hospitals to reduce any bias; however, further investigation is needed, as it is possible that the severity of a patient’s disease may influence the outcomes associated with differing eye drop administration frequency. Second, the duration of the DQSLX treatment was short, at a 4-week treatment. Since dry eye is a chronic disease, long-term studies are required to evaluate the safety and efficacy of DQSLX.

## 5. Conclusions

We demonstrated the efficacy and good patient adherence of a long-acting formulation of diquafosol ophthalmic solution, compared to DQS. DQSLX was more effective than DQS at a lower frequency of administration than DQS. DQSLX and DQS had similar safety profiles.

## Figures and Tables

**Table 1:** Profile of ocular surface and the result of preferred eye drop.

	DQS Group (Pre-Treatment)	DQSLX Group (4 Weeks After Treatment)	*p* Value
Symptoms score	37.8 ± 24.1	23.3 ± 20.1	0.01 *
Preferred eye drops	3/54 (5.6%)	51/54 (94.4%)	<0.001 †
TBUT (s)	4.5 ± 1.7	5.0 ± 2.5	0.75 *
Fluorescein staining score	1.1 ± 1.5	0.8 ± 1.7	<0.001 *

TBUT: tear break-up time. * Wilcoxon signed-rank test. † Fisher’s exact test.

**Table 2:** Profile of ocular surface in subtype of dry eye.

TBUT Subtype		DQS Group (Pre-Treatment)	DQSLX Group (4 Weeks After Treatment)	*p* Value
Random break	Symptoms score	33.3 ± 23.8	20.4 ± 16.8	0.01
(n = 30)	TBUT (s)	5.1 ± 1.5	4.8 ± 2.8	0.32
	Fluorescein staining score	0.8 ± 1.4	0.5 ± 1.5	0.06
Dimple break	Symptoms score	41.9 ± 26.9	22.4 ± 22.4	0.04
(n = 14)	TBUT (s)	4.4 ± 1.6	5.3 ± 2.1	0.67
	Fluorescein staining score	0.4 ± 0.9	0.2 ± 0.8	<0.001
Line break	Symptoms score	45.7 ± 18.7	33.1 ± 23.4	0.11
(n = 10)	TBUT (s)	2.8 ± 0.6	3.3 ± 1.1	0.60
	Fluorescein staining score	0.4 ± 0.9	0.2 ± 0.8	<0.001

Wilcoxon signed-rank test.

## Data Availability

Data are available upon reasonable request.
